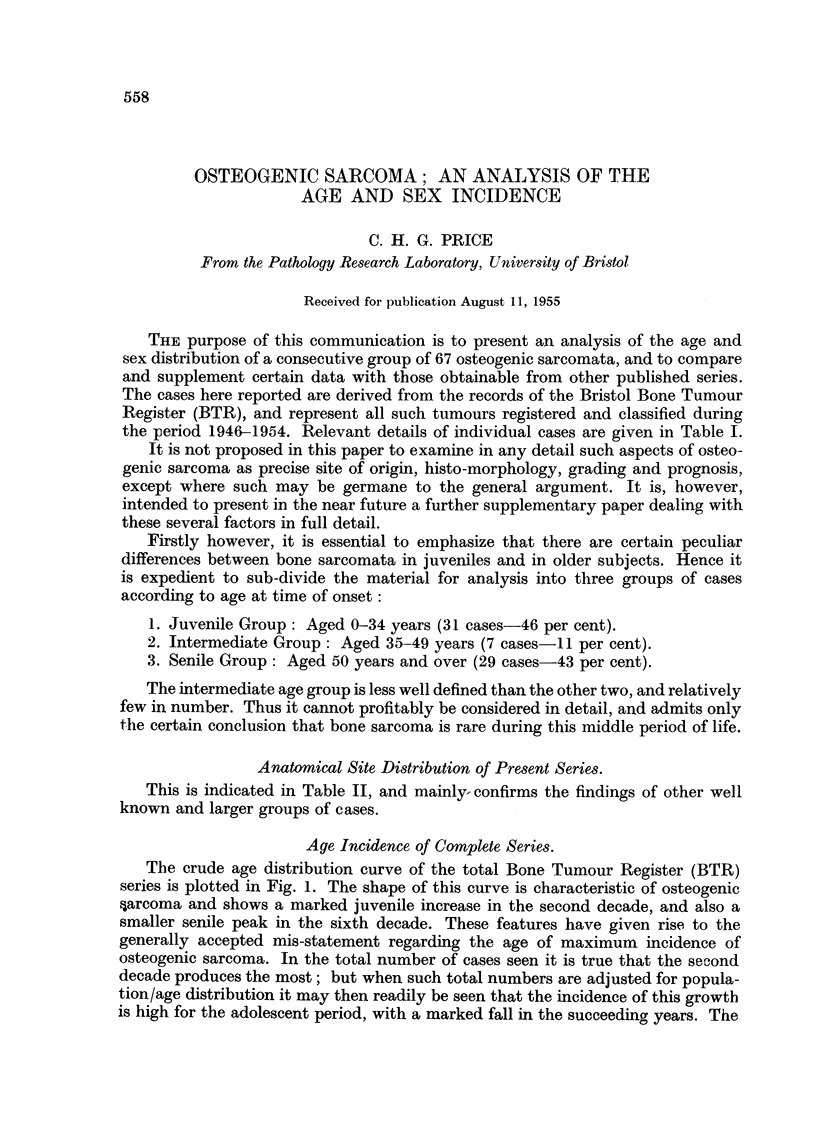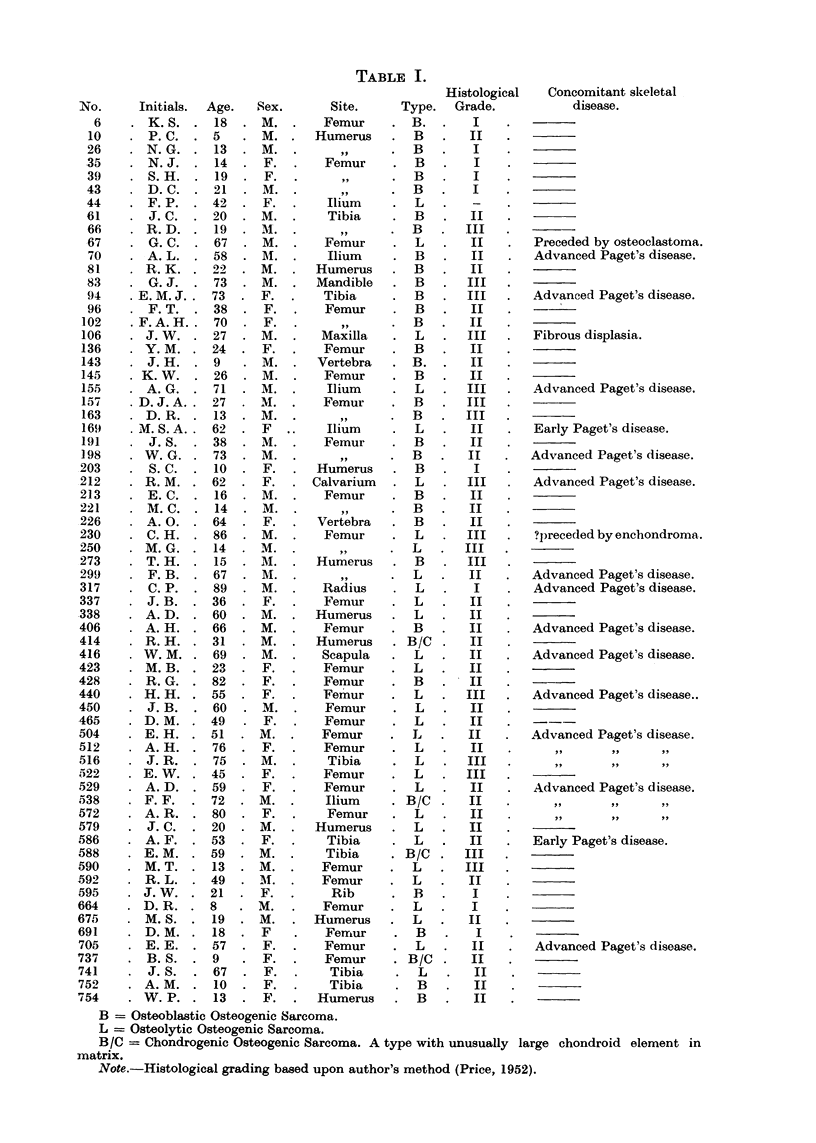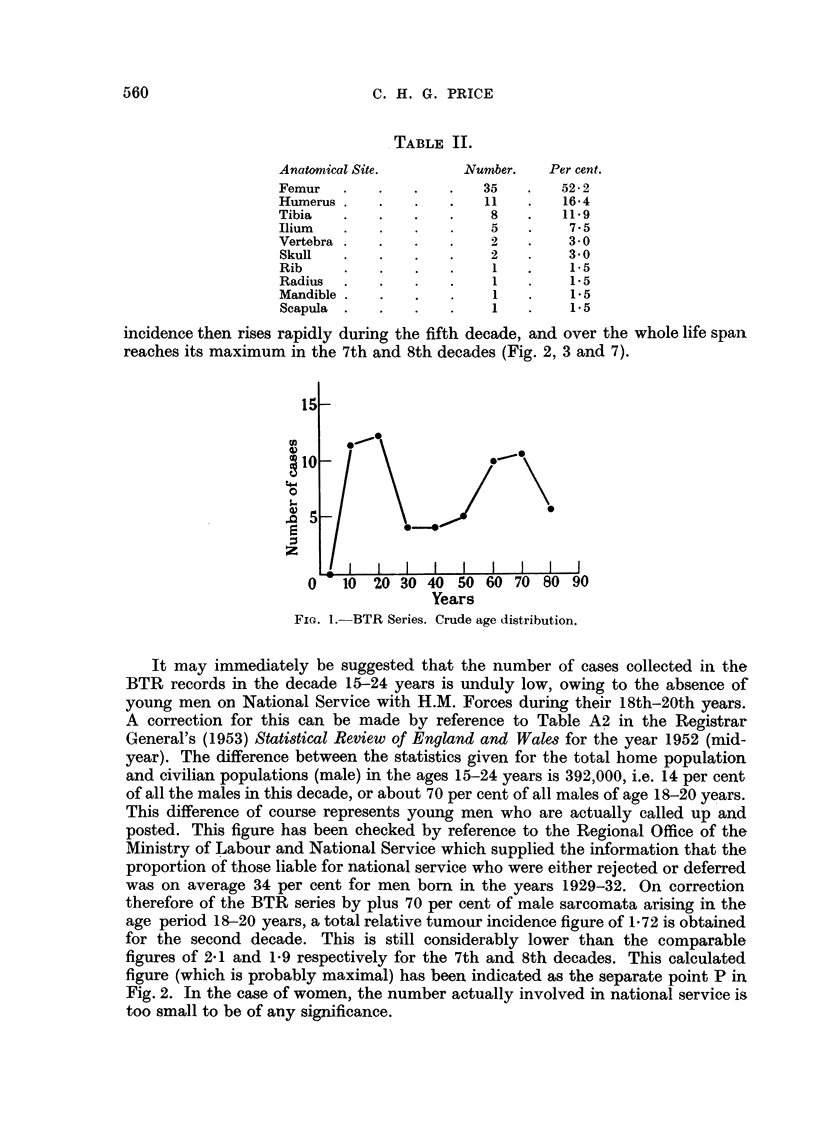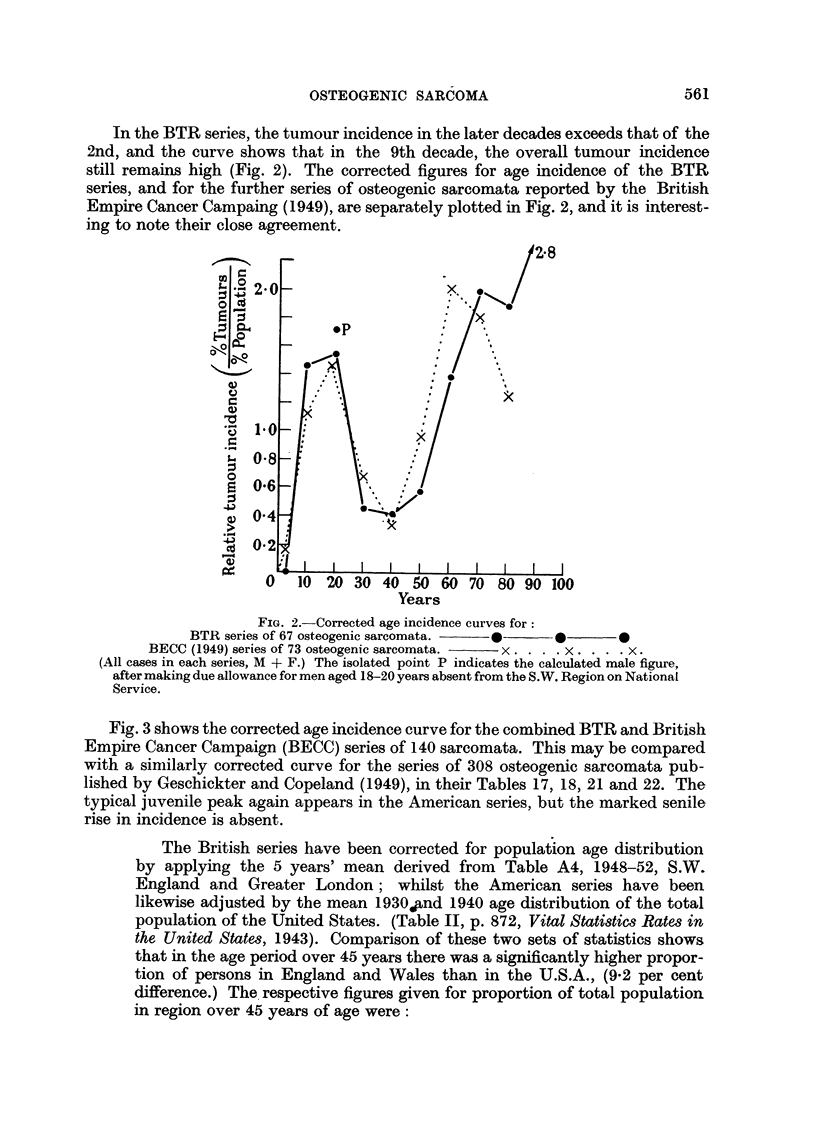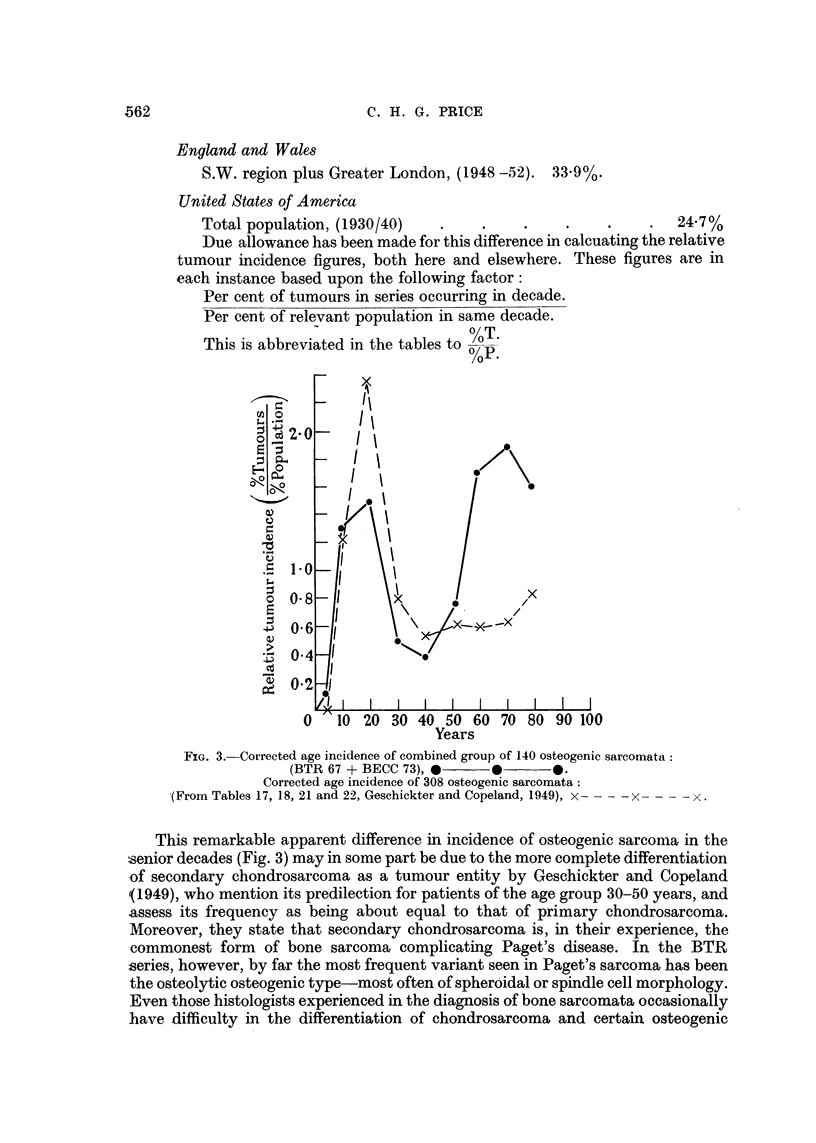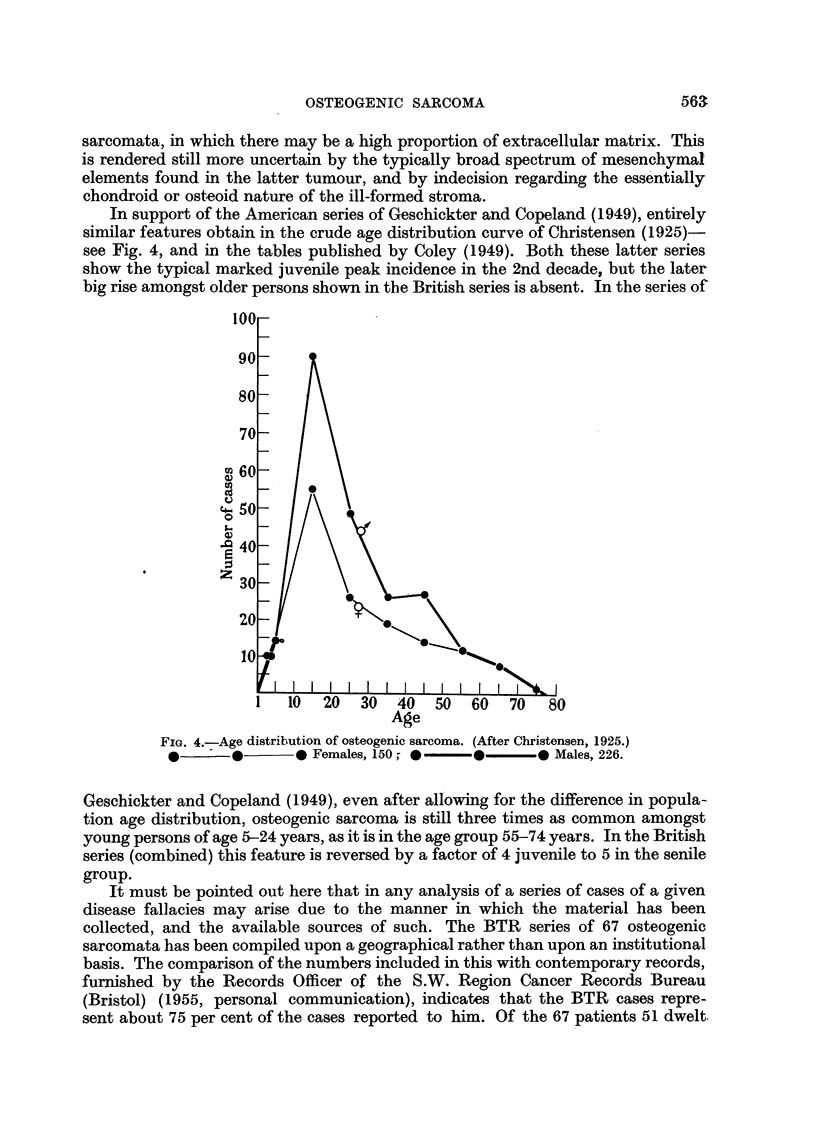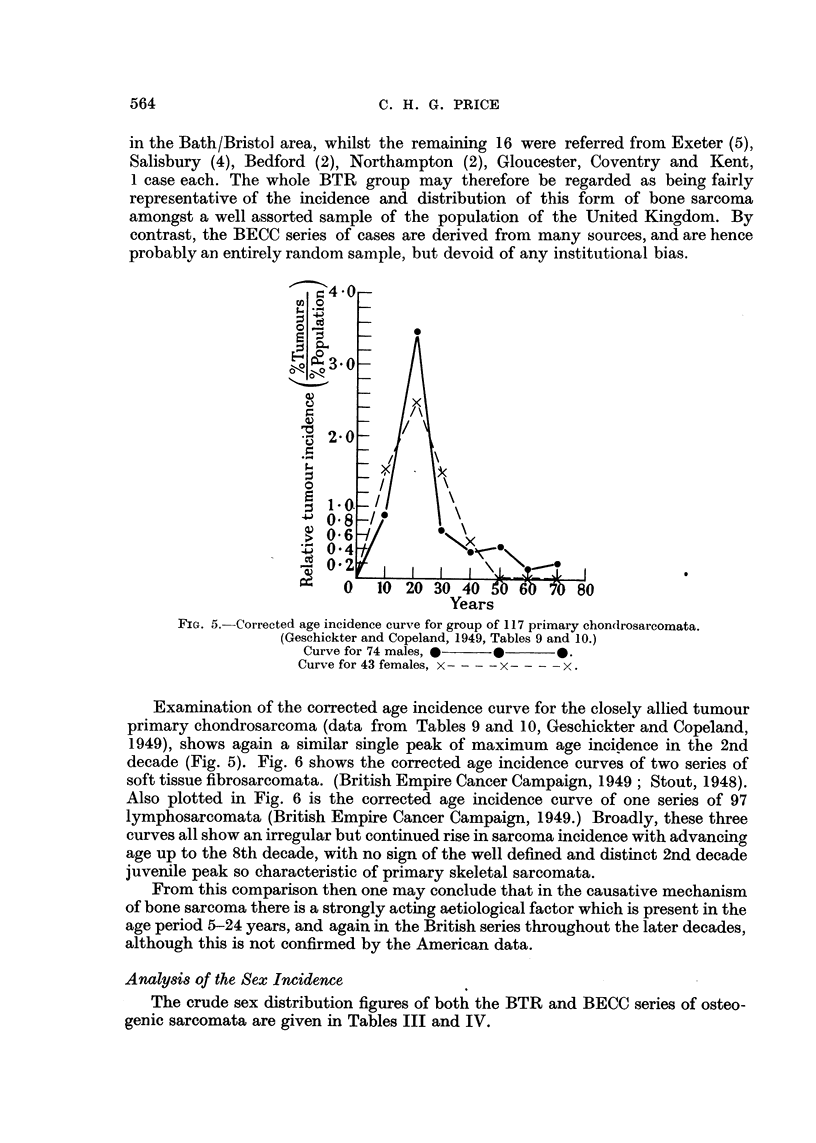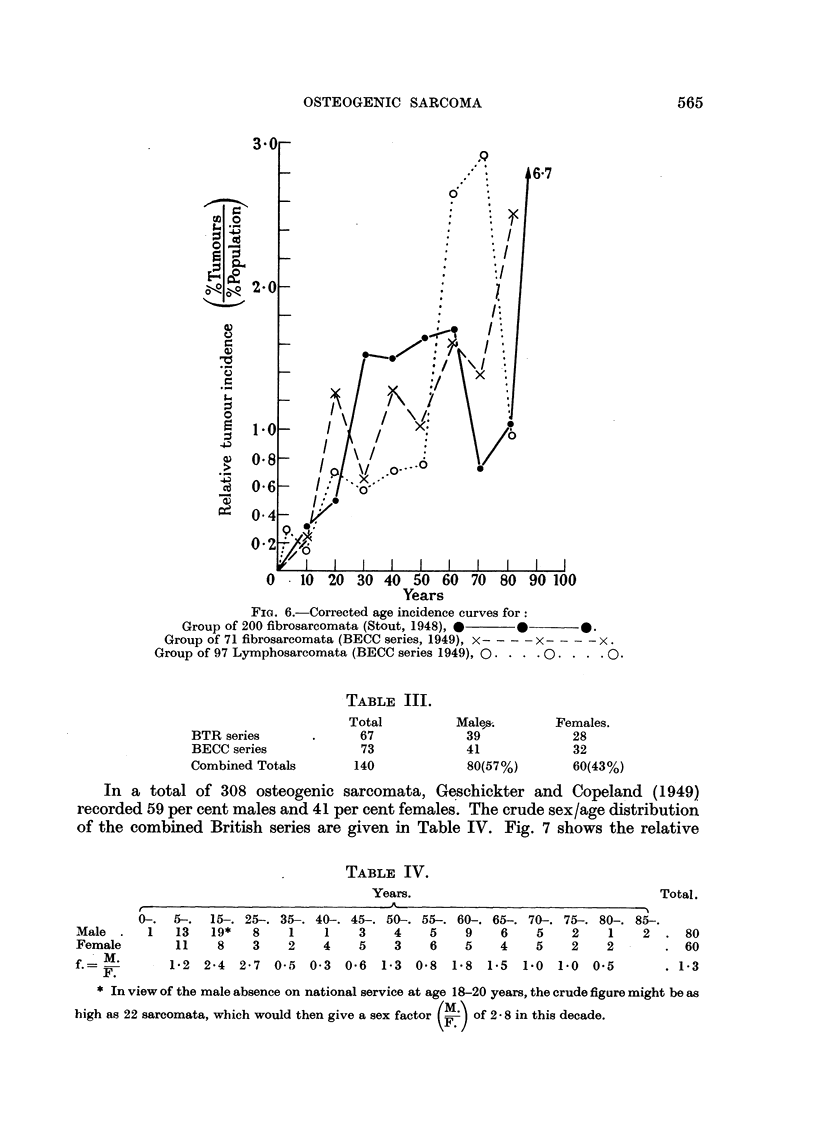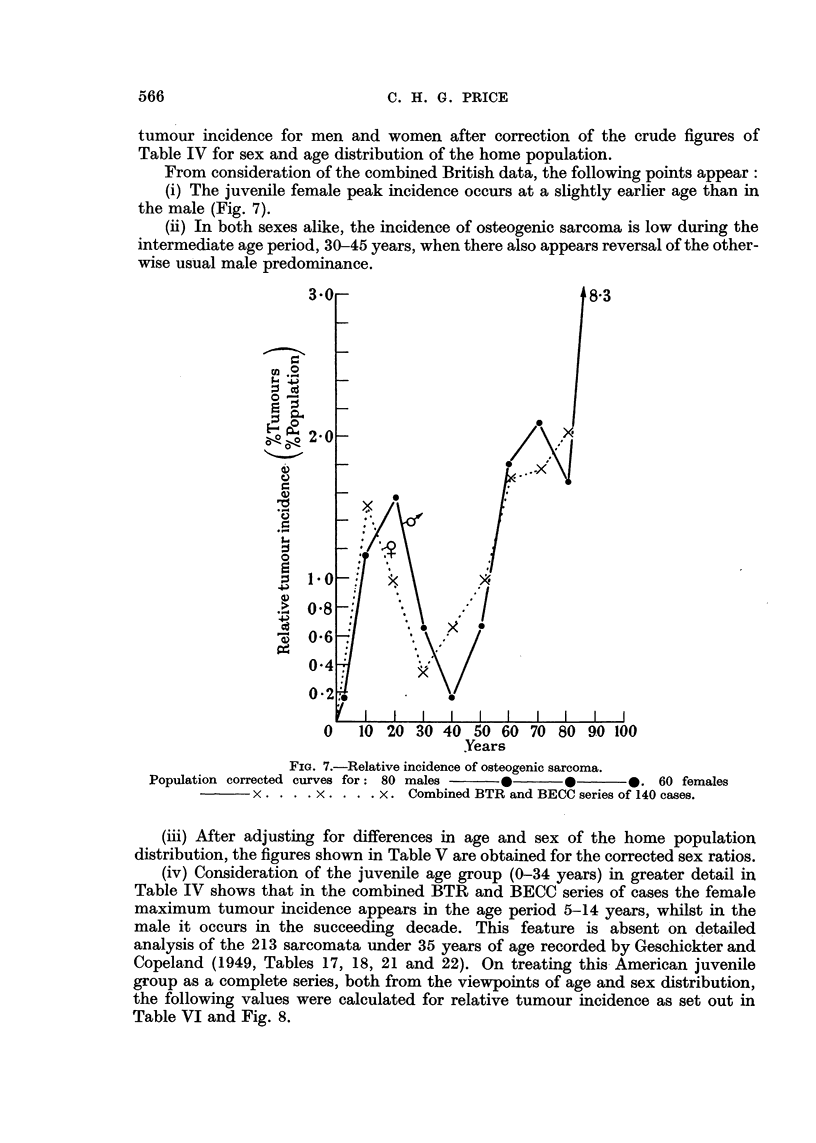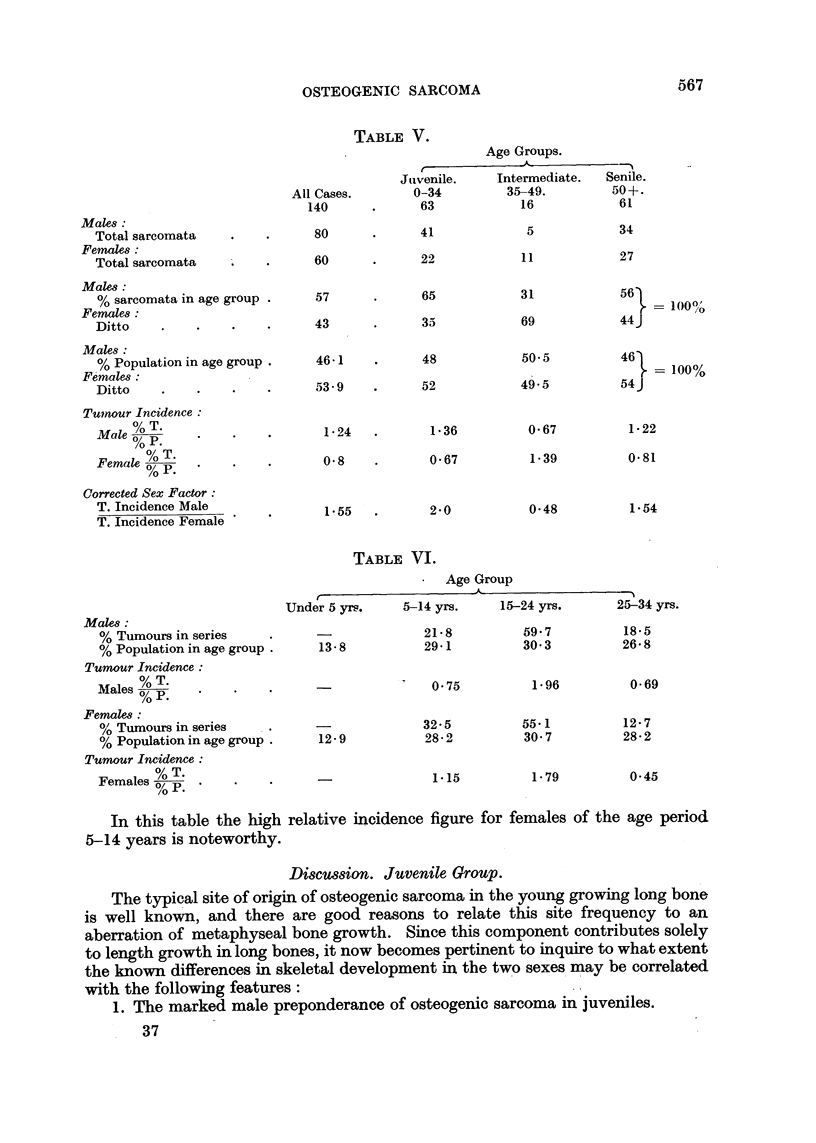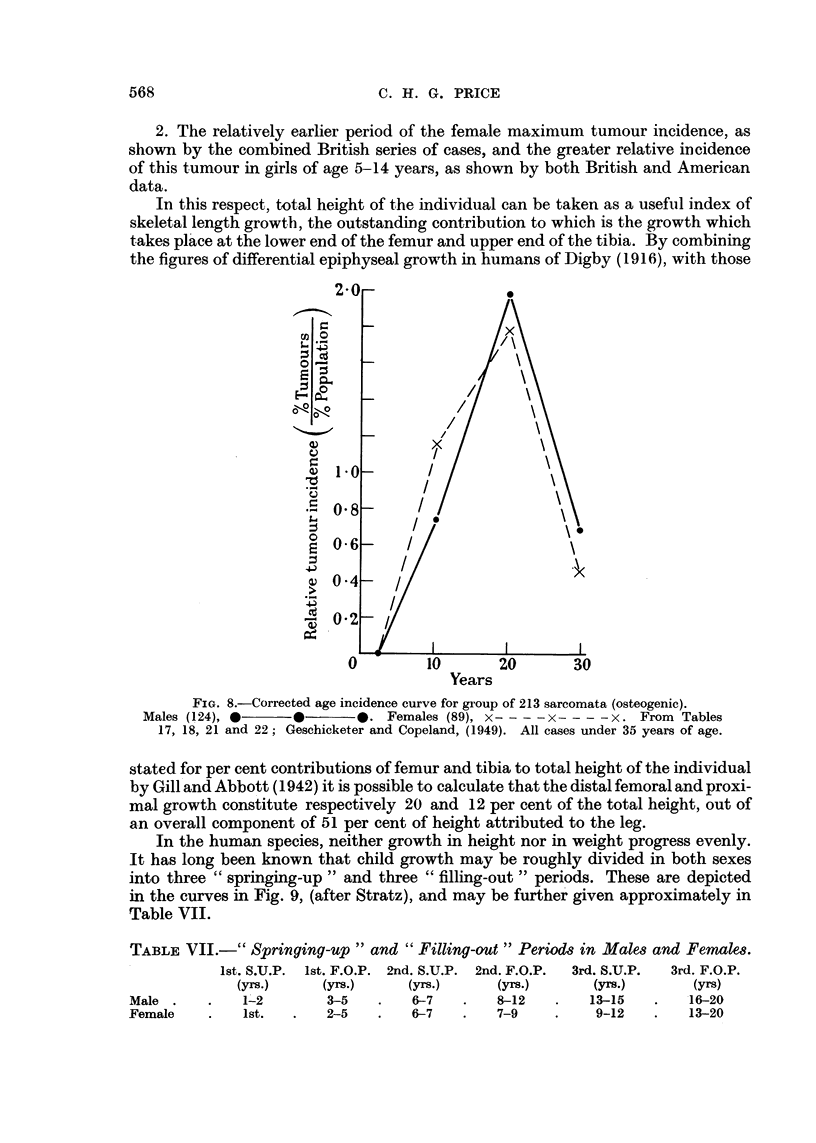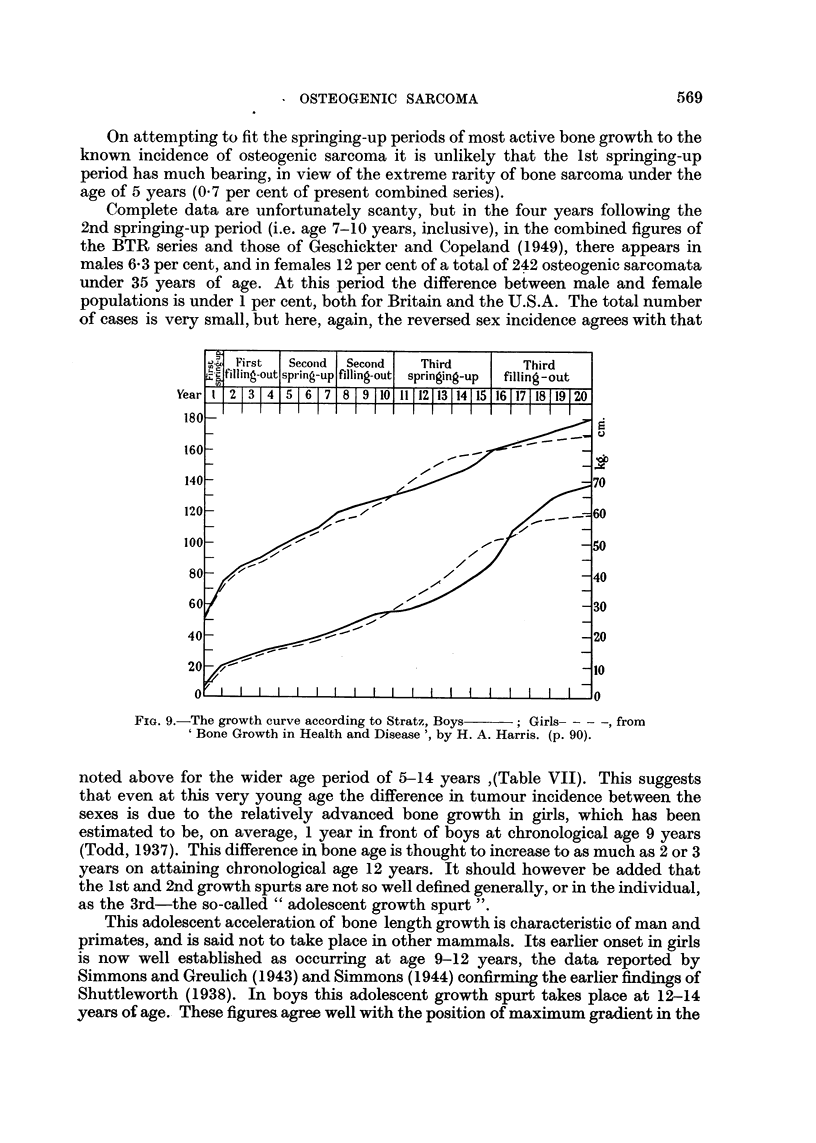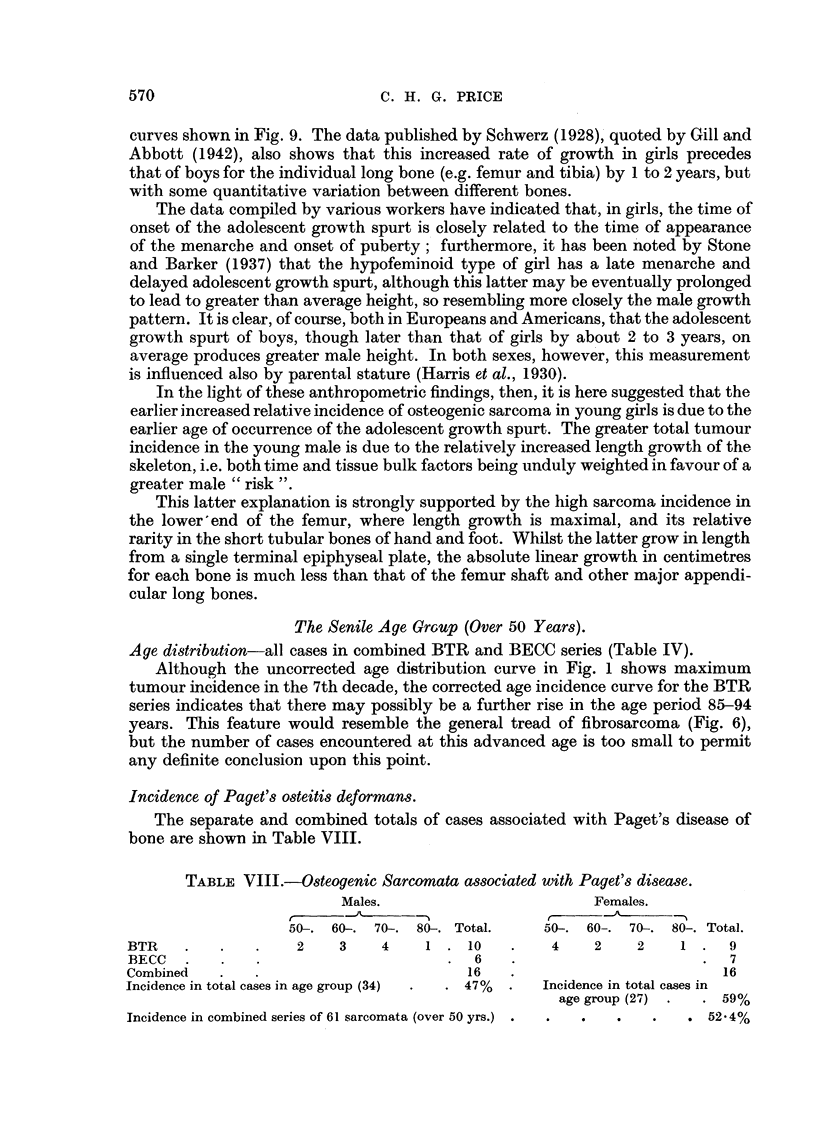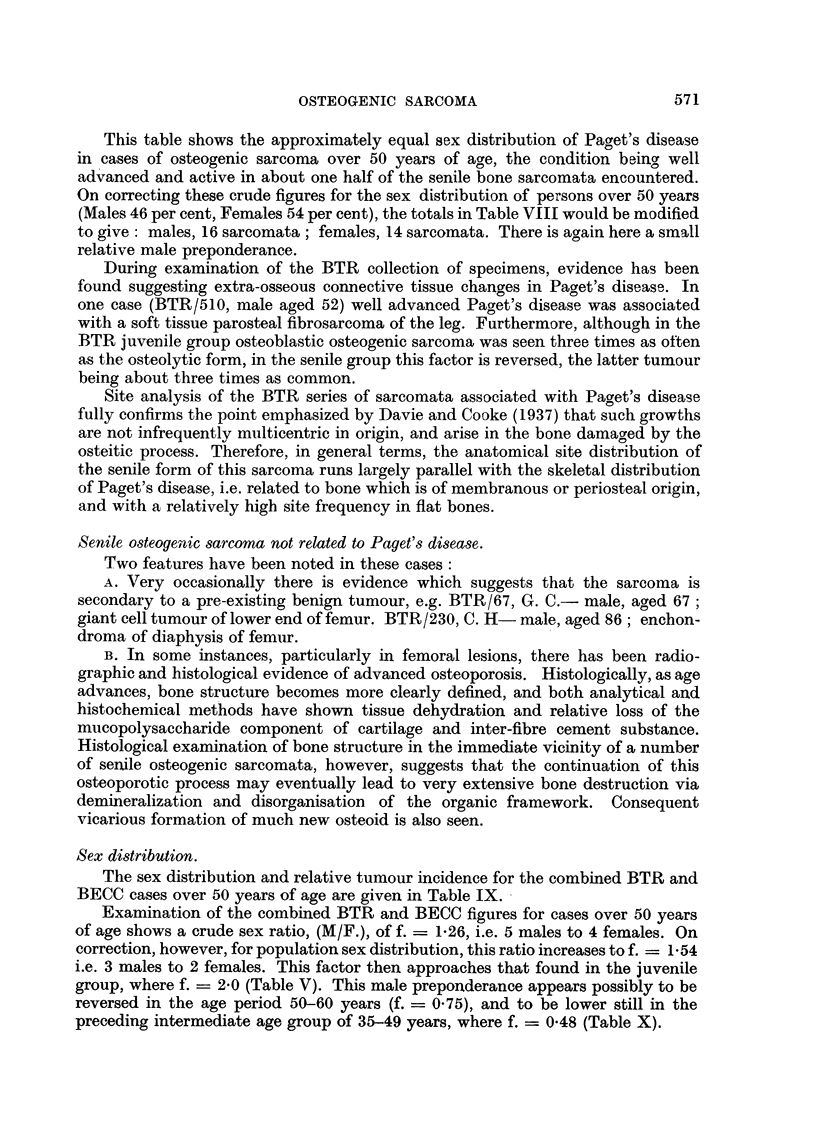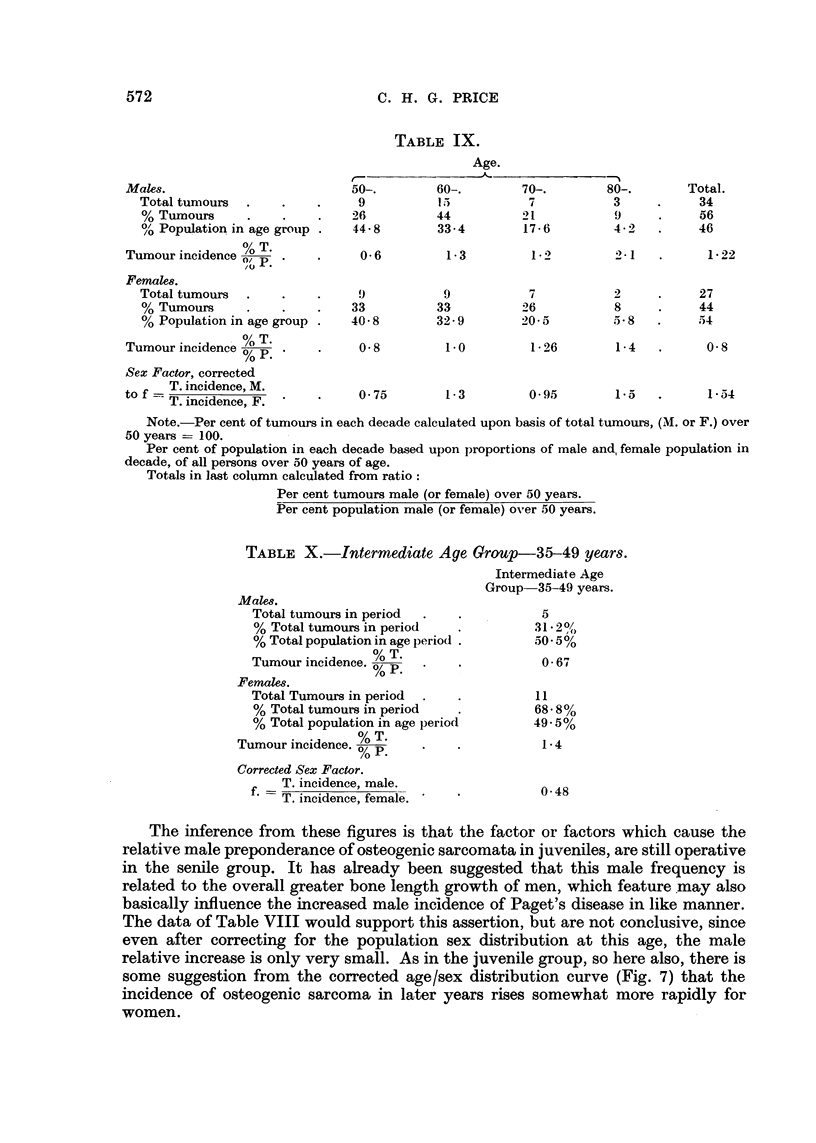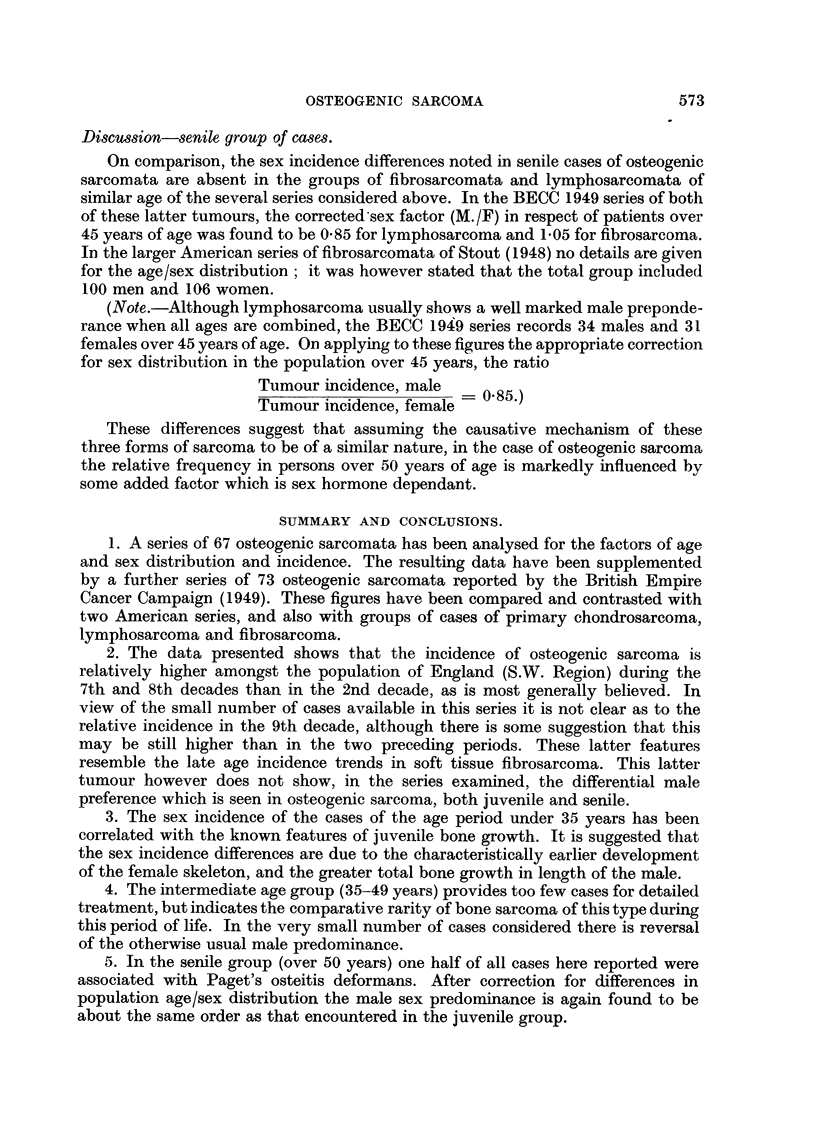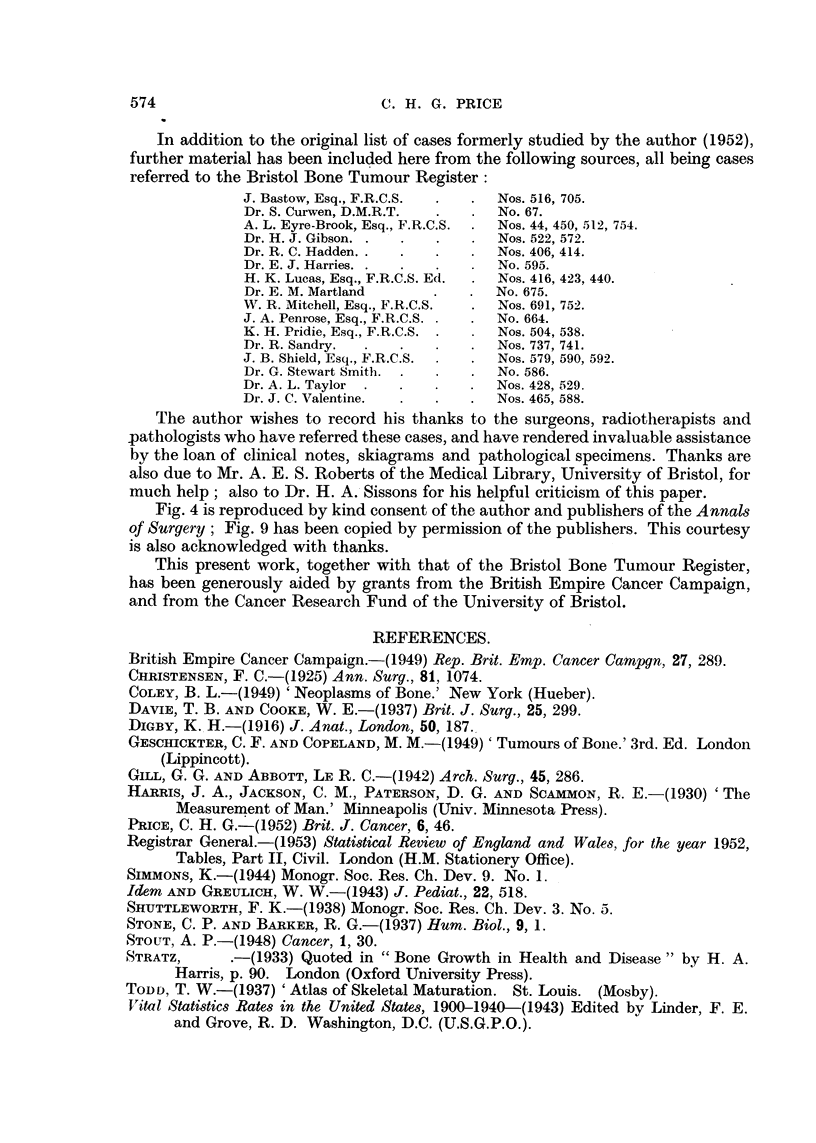# Osteogenic Sarconma; An Analysis of the Age and Sex Incidence

**DOI:** 10.1038/bjc.1955.57

**Published:** 1955-12

**Authors:** C. H. G. Price


### OSTEOGENIC SARCOMA; AN ANALYSIS OF THE AGE AND SEX INCIDENCE

C. H. G. PRICE

*From the Pathology Research Laboratory, University of Bristol*

Received for publication August 11, 1955

THE purpose of this communication is to present an analysis of the age and sex distribution of a consecutive group of 67 osteogenic sarcomata, and to compare and supplement certain data with those obtainable from other published series. The cases here reported are derived from the records of the Bristol Bone Tumour Register (BTR), and represent all such tumours registered and classified during the period 1946–1954. Relevant details of individual cases are given in Table I.

It is not proposed in this paper to examine in any detail such aspects of osteogenic sarcoma as precise site of origin, histo-morphology, grading and prognosis, except where such may be germane to the general argument. It is, however, intended to present in the near future a further supplementary paper dealing with these several factors in full detail.

Firstly however, it is essential to emphasize that there are certain peculiar differences between bone sarcomata in juveniles and in older subjects. Hence it is expedient to sub-divide the material for analysis into three groups of cases according to age at time of onset:

1. Juvenile Group: Aged 0–34 years (31 cases—46 per cent).
2. Intermediate Group: Aged 35–49 years (7 cases—11 per cent).
3. Senile Group: Aged 50 years and over (29 cases—43 per cent).

The intermediate age group is less well defined than the other two, and relatively few in number. Thus it cannot profitably be considered in detail, and admits only the certain conclusion that bone sarcoma is rare during this middle period of life.

### *Anatomical Site Distribution of Present Series.*

This is indicated in Table II, and mainly confirms the findings of other well known and larger groups of cases.

### *Age Incidence of Complete Series.*

The crude age distribution curve of the total Bone Tumour Register (BTR) series is plotted in Fig. 1. The shape of this curve is characteristic of osteogenic sarcoma and shows a marked juvenile increase in the second decade, and also a smaller senile peak in the sixth decade. These features have given rise to the generally accepted mis-statement regarding the age of maximum incidence of osteogenic sarcoma. In the total number of cases seen it is true that the second decade produces the most; but when such total numbers are adjusted for population/age distribution it may then readily be seen that the incidence of this growth is high for the adolescent period, with a marked fall in the succeeding years. The

### Table I.

| No. | Initials. | Age. | Sex. | Site. | Type. | Histological Grade. | Concomitant skeletal disease. |
|---|---|---|---|---|---|---|---|
| 6 | K. S. | 18 | M. | Femur | B. | I | — |
| 10 | P. C. | 5 | M. | Humerus | B | II | — |
| 26 | N. G. | 13 | M. | ,, | B | I | — |
| 35 | N. J. | 14 | F. | Femur | B | I | — |
| 39 | S. H. | 19 | F. | ,, | B | I | — |
| 43 | D. C. | 21 | M. | ,, | B | I | — |
| 44 | F. P. | 42 | F. | Ilium | L | – | — |
| 61 | J. C. | 20 | M. | Tibia | B | II | — |
| 66 | R. D. | 19 | M. | ,, | B | III | — |
| 67 | G. C. | 67 | M. | Femur | L | II | Preceded by osteoclastoma. |
| 70 | A. L. | 58 | M. | Ilium | B | II | Advanced Paget's disease. |
| 81 | R. K. | 22 | M. | Humerus | B | II | — |
| 83 | G. J. | 73 | M. | Mandible | B | III | — |
| 94 | E. M. J. | 73 | F. | Tibia | B | III | Advanced Paget's disease. |
| 96 | F. T. | 38 | F. | Femur | B | II | — |
| 102 | F. A. H. | 70 | F. | ,, | B | II | — |
| 106 | J. W. | 27 | M. | Maxilla | L | III | Fibrous displasia. |
| 136 | Y. M. | 24 | F. | Femur | B | II | — |
| 143 | J. H. | 9 | M. | Vertebra | B. | II | — |
| 145 | K. W. | 26 | M. | Femur | B | II | — |
| 155 | A. G. | 71 | M. | Ilium | L | III | Advanced Paget's disease. |
| 157 | D. J. A. | 27 | M. | Femur | B | III | — |
| 163 | D. R. | 13 | M. | ,, | B | III | — |
| 169 | M. S. A. | 62 | F | Ilium | L | II | Early Paget's disease. |
| 191 | J. S. | 38 | M. | Femur | B | II | — |
| 198 | W. G. | 73 | M. | ,, | B | II | Advanced Paget's disease. |
| 203 | S. C. | 10 | F. | Humerus | B | I | — |
| 212 | R. M. | 62 | F. | Calvarium | L | III | Advanced Paget's disease. |
| 213 | E. C. | 16 | M. | Femur | B | II | — |
| 221 | M. C. | 14 | M. | ,, | B | II | — |
| 226 | A. O. | 64 | F. | Vertebra | B | II | — |
| 230 | C. H. | 86 | M. | Femur | L | III | ?preceded by enchondroma. |
| 250 | M. G. | 14 | M. | ,, | L | III | — |
| 273 | T. H. | 15 | M. | Humerus | B | III | — |
| 299 | F. B. | 67 | M. | ,, | L | II | Advanced Paget's disease. |
| 317 | C. P. | 89 | M. | Radius | L | I | Advanced Paget's disease. |
| 337 | J. B. | 36 | F. | Femur | L | II | — |
| 338 | A. D. | 60 | M. | Humerus | L | II | — |
| 406 | A. H. | 66 | M. | Femur | B | II | Advanced Paget's disease. |
| 414 | R. H. | 31 | M. | Humerus | B/C | II | — |
| 416 | W. M. | 69 | M. | Scapula | L | II | Advanced Paget's disease. |
| 423 | M. B. | 23 | F. | Femur | L | II | — |
| 428 | R. G. | 82 | F. | Femur | B | II | — |
| 440 | H. H. | 55 | F. | Femur | L | III | Advanced Paget's disease.. |
| 450 | J. B. | 60 | M. | Femur | L | II | — |
| 465 | D. M. | 49 | F. | Femur | L | II | — |
| 504 | E. H. | 51 | M. | Femur | L | II | Advanced Paget's disease. |
| 512 | A. H. | 76 | F. | Femur | L | II | ,, ,, ,, |
| 516 | J. R. | 75 | M. | Tibia | L | III | ,, ,, ,, |
| 522 | E. W. | 45 | F. | Femur | L | III | — |
| 529 | A. D. | 59 | F. | Femur | L | II | Advanced Paget's disease. |
| 538 | F. F. | 72 | M. | Ilium | B/C | II | ,, ,, ,, |
| 572 | A. R. | 80 | F. | Femur | L | II | ,, ,, ,, |
| 579 | J. C. | 20 | M. | Humerus | L | II | — |
| 586 | A. F. | 53 | F. | Tibia | L | II | Early Paget's disease. |
| 588 | E. M. | 59 | M. | Tibia | B/C | III | — |
| 590 | M. T. | 13 | M. | Femur | L | III | — |
| 592 | R. L. | 49 | M. | Femur | L | II | — |
| 595 | J. W. | 21 | F. | Rib | B | I | — |
| 664 | D. R. | 8 | M. | Femur | L | I | — |
| 675 | M. S. | 19 | M. | Humerus | L | II | — |
| 691 | D. M. | 18 | F | Femur | B | I | — |
| 705 | E. E. | 57 | F. | Femur | L | II | Advanced Paget's disease. |
| 737 | B. S. | 9 | F. | Femur | B/C | II | — |
| 741 | J. S. | 67 | F. | Tibia | L | II | — |
| 752 | A. M. | 10 | F. | Tibia | B | II | — |
| 754 | W. P. | 13 | F. | Humerus | B | II | — |

B = Osteoblastic Osteogenic Sarcoma.
L = Osteolytic Osteogenic Sarcoma.
B/C = Chondrogenic Osteogenic Sarcoma. A type with unusually large chondroid element in matrix.

*Note*.—Histological grading based upon author's method (Price, 1952).

TABLE II.

| *Anatomical Site.* | *Number.* | *Per cent.* |
|---|---|---|
| Femur | 35 | 52·2 |
| Humerus | 11 | 16·4 |
| Tibia | 8 | 11·9 |
| Ilium | 5 | 7·5 |
| Vertebra | 2 | 3·0 |
| Skull | 2 | 3·0 |
| Rib | 1 | 1·5 |
| Radius | 1 | 1·5 |
| Mandible | 1 | 1·5 |
| Scapula | 1 | 1·5 |

incidence then rises rapidly during the fifth decade, and over the whole life span reaches its maximum in the 7th and 8th decades (Fig. 2, 3 and 7).

FIG. 1.—BTR Series. Crude age distribution.

It may immediately be suggested that the number of cases collected in the BTR records in the decade 15–24 years is unduly low, owing to the absence of young men on National Service with H.M. Forces during their 18th–20th years. A correction for this can be made by reference to Table A2 in the Registrar General's (1953) *Statistical Review of England and Wales* for the year 1952 (mid-year). The difference between the statistics given for the total home population and civilian populations (male) in the ages 15–24 years is 392,000, i.e. 14 per cent of all the males in this decade, or about 70 per cent of all males of age 18–20 years. This difference of course represents young men who are actually called up and posted. This figure has been checked by reference to the Regional Office of the Ministry of Labour and National Service which supplied the information that the proportion of those liable for national service who were either rejected or deferred was on average 34 per cent for men born in the years 1929–32. On correction therefore of the BTR series by plus 70 per cent of male sarcomata arising in the age period 18–20 years, a total relative tumour incidence figure of 1·72 is obtained for the second decade. This is still considerably lower than the comparable figures of 2·1 and 1·9 respectively for the 7th and 8th decades. This calculated figure (which is probably maximal) has been indicated as the separate point P in Fig. 2. In the case of women, the number actually involved in national service is too small to be of any significance.

In the BTR series, the tumour incidence in the later decades exceeds that of the 2nd, and the curve shows that in the 9th decade, the overall tumour incidence still remains high (Fig. 2). The corrected figures for age incidence of the BTR series, and for the further series of osteogenic sarcomata reported by the British Empire Cancer Campaing (1949), are separately plotted in Fig. 2, and it is interesting to note their close agreement.

FIG. 2.—Corrected age incidence curves for :
BTR series of 67 osteogenic sarcomata. ——●——●——●
BECC (1949) series of 73 osteogenic sarcomata. ——×. . . . .×. . . . .×.
(All cases in each series, M + F.) The isolated point P indicates the calculated male figure, after making due allowance for men aged 18–20 years absent from the S.W. Region on National Service.

Fig. 3 shows the corrected age incidence curve for the combined BTR and British Empire Cancer Campaign (BECC) series of 140 sarcomata. This may be compared with a similarly corrected curve for the series of 308 osteogenic sarcomata published by Geschickter and Copeland (1949), in their Tables 17, 18, 21 and 22. The typical juvenile peak again appears in the American series, but the marked senile rise in incidence is absent.

The British series have been corrected for population age distribution by applying the 5 years' mean derived from Table A4, 1948–52, S.W. England and Greater London ; whilst the American series have been likewise adjusted by the mean 1930 and 1940 age distribution of the total population of the United States. (Table II, p. 872, *Vital Statistics Rates in the United States*, 1943). Comparison of these two sets of statistics shows that in the age period over 45 years there was a significantly higher proportion of persons in England and Wales than in the U.S.A., (9·2 per cent difference.) The respective figures given for proportion of total population in region over 45 years of age were :

*England and Wales*

S.W. region plus Greater London, (1948–52). 33·9%.

*United States of America*

Total population, (1930/40) . . . . . . . 24·7%

Due allowance has been made for this difference in calcuating the relative tumour incidence figures, both here and elsewhere. These figures are in each instance based upon the following factor :

$$\frac{\text{Per cent of tumours in series occurring in decade.}}{\text{Per cent of relevant population in same decade.}}$$

This is abbreviated in the tables to $\frac{\%T.}{\%P.}$

FIG. 3.—Corrected age incidence of combined group of 140 osteogenic sarcomata : (BTR 67 + BECC 73), ●———●———●.
Corrected age incidence of 308 osteogenic sarcomata :
(From Tables 17, 18, 21 and 22, Geschickter and Copeland, 1949), ×- - - -×- - - -×.

This remarkable apparent difference in incidence of osteogenic sarcoma in the senior decades (Fig. 3) may in some part be due to the more complete differentiation of secondary chondrosarcoma as a tumour entity by Geschickter and Copeland (1949), who mention its predilection for patients of the age group 30–50 years, and assess its frequency as being about equal to that of primary chondrosarcoma. Moreover, they state that secondary chondrosarcoma is, in their experience, the commonest form of bone sarcoma complicating Paget's disease. In the BTR series, however, by far the most frequent variant seen in Paget's sarcoma has been the osteolytic osteogenic type—most often of spheroidal or spindle cell morphology. Even those histologists experienced in the diagnosis of bone sarcomata occasionally have difficulty in the differentiation of chondrosarcoma and certain osteogenic sarcomata, in which there may be a high proportion of extracellular matrix. This is rendered still more uncertain by the typically broad spectrum of mesenchymal elements found in the latter tumour, and by indecision regarding the essentially chondroid or osteoid nature of the ill-formed stroma.

In support of the American series of Geschickter and Copeland (1949), entirely similar features obtain in the crude age distribution curve of Christensen (1925)—see Fig. 4, and in the tables published by Coley (1949). Both these latter series show the typical marked juvenile peak incidence in the 2nd decade, but the later big rise amongst older persons shown in the British series is absent. In the series of

FIG. 4.—Age distribution of osteogenic sarcoma. (After Christensen, 1925.) ●———●———● Females, 150; ●———●———● Males, 226.

Geschickter and Copeland (1949), even after allowing for the difference in population age distribution, osteogenic sarcoma is still three times as common amongst young persons of age 5–24 years, as it is in the age group 55–74 years. In the British series (combined) this feature is reversed by a factor of 4 juvenile to 5 in the senile group.

It must be pointed out here that in any analysis of a series of cases of a given disease fallacies may arise due to the manner in which the material has been collected, and the available sources of such. The BTR series of 67 osteogenic sarcomata has been compiled upon a geographical rather than upon an institutional basis. The comparison of the numbers included in this with contemporary records, furnished by the Records Officer of the S.W. Region Cancer Records Bureau (Bristol) (1955, personal communication), indicates that the BTR cases represent about 75 per cent of the cases reported to him. Of the 67 patients 51 dwelt in the Bath/Bristol area, whilst the remaining 16 were referred from Exeter (5), Salisbury (4), Bedford (2), Northampton (2), Gloucester, Coventry and Kent, 1 case each. The whole BTR group may therefore be regarded as being fairly representative of the incidence and distribution of this form of bone sarcoma amongst a well assorted sample of the population of the United Kingdom. By contrast, the BECC series of cases are derived from many sources, and are hence probably an entirely random sample, but devoid of any institutional bias.

FIG. 5.—Corrected age incidence curve for group of 117 primary chondrosarcomata. (Geschickter and Copeland, 1949, Tables 9 and 10.)
Curve for 74 males, ●———●———●.
Curve for 43 females, ×- - - -×- - - -×.

Examination of the corrected age incidence curve for the closely allied tumour primary chondrosarcoma (data from Tables 9 and 10, Geschickter and Copeland, 1949), shows again a similar single peak of maximum age incidence in the 2nd decade (Fig. 5). Fig. 6 shows the corrected age incidence curves of two series of soft tissue fibrosarcomata. (British Empire Cancer Campaign, 1949 ; Stout, 1948). Also plotted in Fig. 6 is the corrected age incidence curve of one series of 97 lymphosarcomata (British Empire Cancer Campaign, 1949.) Broadly, these three curves all show an irregular but continued rise in sarcoma incidence with advancing age up to the 8th decade, with no sign of the well defined and distinct 2nd decade juvenile peak so characteristic of primary skeletal sarcomata.

From this comparison then one may conclude that in the causative mechanism of bone sarcoma there is a strongly acting aetiological factor which is present in the age period 5–24 years, and again in the British series throughout the later decades, although this is not confirmed by the American data.

### *Analysis of the Sex Incidence*

The crude sex distribution figures of both the BTR and BECC series of osteogenic sarcomata are given in Tables III and IV.

FIG. 6.—Corrected age incidence curves for :
Group of 200 fibrosarcomata (Stout, 1948), ●————●————●.
Group of 71 fibrosarcomata (BECC series, 1949), ×– – – –×– – – –×.
Group of 97 Lymphosarcomata (BECC series 1949), ○. . . .○. . . .○.

TABLE III.

| | Total | Males. | Females. |
|---|---|---|---|
| BTR series . | 67 | 39 | 28 |
| BECC series | 73 | 41 | 32 |
| Combined Totals | 140 | 80(57%) | 60(43%) |

In a total of 308 osteogenic sarcomata, Geschickter and Copeland (1949) recorded 59 per cent males and 41 per cent females. The crude sex/age distribution of the combined British series are given in Table IV. Fig. 7 shows the relative

TABLE IV.

| | Years. | | | | | | | | | | | | | | | Total. |
|---|---|---|---|---|---|---|---|---|---|---|---|---|---|---|---|---|
| | 0–. | 5–. | 15–. | 25–. | 35–. | 40–. | 45–. | 50–. | 55–. | 60–. | 65–. | 70–. | 75–. | 80–. | 85–. | |
| Male . | 1 | 13 | 19* | 8 | 1 | 1 | 3 | 4 | 5 | 9 | 6 | 5 | 2 | 1 | 2 | . 80 |
| Female | | 11 | 8 | 3 | 2 | 4 | 5 | 3 | 6 | 5 | 4 | 5 | 2 | 2 | | . 60 |
| f. = $\frac{M.}{F.}$ | | 1·2 | 2·4 | 2·7 | 0·5 | 0·3 | 0·6 | 1·3 | 0·8 | 1·8 | 1·5 | 1·0 | 1·0 | 0·5 | | . 1·3 |

* In view of the male absence on national service at age 18–20 years, the crude figure might be as high as 22 sarcomata, which would then give a sex factor $\left(\frac{M.}{F.}\right)$ of 2·8 in this decade.

tumour incidence for men and women after correction of the crude figures of Table IV for sex and age distribution of the home population.

From consideration of the combined British data, the following points appear :

(i) The juvenile female peak incidence occurs at a slightly earlier age than in the male (Fig. 7).

(ii) In both sexes alike, the incidence of osteogenic sarcoma is low during the intermediate age period, 30–45 years, when there also appears reversal of the otherwise usual male predominance.

FIG. 7.—Relative incidence of osteogenic sarcoma.
Population corrected curves for : 80 males ——●——●——●. 60 females ——×. . . . ×. . . . ×. Combined BTR and BECC series of 140 cases.

(iii) After adjusting for differences in age and sex of the home population distribution, the figures shown in Table V are obtained for the corrected sex ratios.

(iv) Consideration of the juvenile age group (0–34 years) in greater detail in Table IV shows that in the combined BTR and BECC series of cases the female maximum tumour incidence appears in the age period 5–14 years, whilst in the male it occurs in the succeeding decade. This feature is absent on detailed analysis of the 213 sarcomata under 35 years of age recorded by Geschickter and Copeland (1949, Tables 17, 18, 21 and 22). On treating this American juvenile group as a complete series, both from the viewpoints of age and sex distribution, the following values were calculated for relative tumour incidence as set out in Table VI and Fig. 8.

TABLE V.

| | All Cases. | Age Groups. Juvenile. 0–34 | Intermediate. 35–49. | Senile. 50+. | |
|---|---|---|---|---|---|
| | 140 | 63 | 16 | 61 | |
| *Males :* Total sarcomata | 80 | 41 | 5 | 34 | |
| *Females :* Total sarcomata | 60 | 22 | 11 | 27 | |
| *Males :* % sarcomata in age group | 57 | 65 | 31 | 56 | = 100% |
| *Females :* Ditto | 43 | 35 | 69 | 44 | |
| *Males :* % Population in age group | 46·1 | 48 | 50·5 | 46 | = 100% |
| *Females :* Ditto | 53·9 | 52 | 49·5 | 54 | |
| *Tumour Incidence :* | | | | | |
| *Male* $\frac{\% \text{T.}}{\% \text{P.}}$ | 1·24 | 1·36 | 0·67 | 1·22 | |
| *Female* $\frac{\% \text{T.}}{\% \text{P.}}$ | 0·8 | 0·67 | 1·39 | 0·81 | |
| *Corrected Sex Factor :* $\frac{\text{T. Incidence Male}}{\text{T. Incidence Female}}$ | 1·55 | 2·0 | 0·48 | 1·54 | |

TABLE VI.

| | Age Group. Under 5 yrs. | 5–14 yrs. | 15–24 yrs. | 25–34 yrs. |
|---|---|---|---|---|
| *Males :* | | | | |
| % Tumours in series | — | 21·8 | 59·7 | 18·5 |
| % Population in age group | 13·8 | 29·1 | 30·3 | 26·8 |
| *Tumour Incidence :* Males $\frac{\% \text{T.}}{\% \text{P.}}$ | — | 0·75 | 1·96 | 0·69 |
| *Females :* | | | | |
| % Tumours in series | — | 32·5 | 55·1 | 12·7 |
| % Population in age group | 12·9 | 28·2 | 30·7 | 28·2 |
| *Tumour Incidence :* Females $\frac{\% \text{T.}}{\% \text{P.}}$ | — | 1·15 | 1·79 | 0·45 |

In this table the high relative incidence figure for females of the age period 5–14 years is noteworthy.

*Discussion. Juvenile Group.*

The typical site of origin of osteogenic sarcoma in the young growing long bone is well known, and there are good reasons to relate this site frequency to an aberration of metaphyseal bone growth. Since this component contributes solely to length growth in long bones, it now becomes pertinent to inquire to what extent the known differences in skeletal development in the two sexes may be correlated with the following features :

1. The marked male preponderance of osteogenic sarcoma in juveniles.

2. The relatively earlier period of the female maximum tumour incidence, as shown by the combined British series of cases, and the greater relative incidence of this tumour in girls of age 5–14 years, as shown by both British and American data.

In this respect, total height of the individual can be taken as a useful index of skeletal length growth, the outstanding contribution to which is the growth which takes place at the lower end of the femur and upper end of the tibia. By combining the figures of differential epiphyseal growth in humans of Digby (1916), with those

FIG. 8.—Corrected age incidence curve for group of 213 sarcomata (osteogenic). Males (124), ●———●———●. Females (89), ×- - - -×- - - -×. From Tables 17, 18, 21 and 22; Geschicketer and Copeland, (1949). All cases under 35 years of age.

stated for per cent contributions of femur and tibia to total height of the individual by Gill and Abbott (1942) it is possible to calculate that the distal femoral and proximal growth constitute respectively 20 and 12 per cent of the total height, out of an overall component of 51 per cent of height attributed to the leg.

In the human species, neither growth in height nor in weight progress evenly. It has long been known that child growth may be roughly divided in both sexes into three " springing-up " and three " filling-out " periods. These are depicted in the curves in Fig. 9, (after Stratz), and may be further given approximately in Table VII.

TABLE VII.—*" Springing-up " and " Filling-out " Periods in Males and Females.*

| | 1st. S.U.P. (yrs.) | 1st. F.O.P. (yrs.) | 2nd. S.U.P. (yrs.) | 2nd. F.O.P. (yrs.) | 3rd. S.U.P. (yrs.) | 3rd. F.O.P. (yrs) |
|---|---|---|---|---|---|---|
| Male | 1–2 | 3–5 | 6–7 | 8–12 | 13–15 | 16–20 |
| Female | 1st. | 2–5 | 6–7 | 7–9 | 9–12 | 13–20 |

On attempting to fit the springing-up periods of most active bone growth to the known incidence of osteogenic sarcoma it is unlikely that the 1st springing-up period has much bearing, in view of the extreme rarity of bone sarcoma under the age of 5 years (0·7 per cent of present combined series).

Complete data are unfortunately scanty, but in the four years following the 2nd springing-up period (i.e. age 7–10 years, inclusive), in the combined figures of the BTR series and those of Geschickter and Copeland (1949), there appears in males 6·3 per cent, and in females 12 per cent of a total of 242 osteogenic sarcomata under 35 years of age. At this period the difference between male and female populations is under 1 per cent, both for Britain and the U.S.A. The total number of cases is very small, but here, again, the reversed sex incidence agrees with that

FIG. 9.—The growth curve according to Stratz, Boys———— ; Girls– – – –, from ' Bone Growth in Health and Disease ', by H. A. Harris. (p. 90).

noted above for the wider age period of 5–14 years ,(Table VII). This suggests that even at this very young age the difference in tumour incidence between the sexes is due to the relatively advanced bone growth in girls, which has been estimated to be, on average, 1 year in front of boys at chronological age 9 years (Todd, 1937). This difference in bone age is thought to increase to as much as 2 or 3 years on attaining chronological age 12 years. It should however be added that the 1st and 2nd growth spurts are not so well defined generally, or in the individual, as the 3rd—the so-called " adolescent growth spurt ".

This adolescent acceleration of bone length growth is characteristic of man and primates, and is said not to take place in other mammals. Its earlier onset in girls is now well established as occurring at age 9–12 years, the data reported by Simmons and Greulich (1943) and Simmons (1944) confirming the earlier findings of Shuttleworth (1938). In boys this adolescent growth spurt takes place at 12–14 years of age. These figures agree well with the position of maximum gradient in the curves shown in Fig. 9. The data published by Schwerz (1928), quoted by Gill and Abbott (1942), also shows that this increased rate of growth in girls precedes that of boys for the individual long bone (e.g. femur and tibia) by 1 to 2 years, but with some quantitative variation between different bones.

The data compiled by various workers have indicated that, in girls, the time of onset of the adolescent growth spurt is closely related to the time of appearance of the menarche and onset of puberty ; furthermore, it has been noted by Stone and Barker (1937) that the hypofeminoid type of girl has a late menarche and delayed adolescent growth spurt, although this latter may be eventually prolonged to lead to greater than average height, so resembling more closely the male growth pattern. It is clear, of course, both in Europeans and Americans, that the adolescent growth spurt of boys, though later than that of girls by about 2 to 3 years, on average produces greater male height. In both sexes, however, this measurement is influenced also by parental stature (Harris *et al.*, 1930).

In the light of these anthropometric findings, then, it is here suggested that the earlier increased relative incidence of osteogenic sarcoma in young girls is due to the earlier age of occurrence of the adolescent growth spurt. The greater total tumour incidence in the young male is due to the relatively increased length growth of the skeleton, i.e. both time and tissue bulk factors being unduly weighted in favour of a greater male " risk ".

This latter explanation is strongly supported by the high sarcoma incidence in the lower end of the femur, where length growth is maximal, and its relative rarity in the short tubular bones of hand and foot. Whilst the latter grow in length from a single terminal epiphyseal plate, the absolute linear growth in centimetres for each bone is much less than that of the femur shaft and other major appendicular long bones.

### *The Senile Age Group (Over 50 Years).*

*Age distribution*—all cases in combined BTR and BECC series (Table IV).

Although the uncorrected age distribution curve in Fig. 1 shows maximum tumour incidence in the 7th decade, the corrected age incidence curve for the BTR series indicates that there may possibly be a further rise in the age period 85–94 years. This feature would resemble the general tread of fibrosarcoma (Fig. 6), but the number of cases encountered at this advanced age is too small to permit any definite conclusion upon this point.

*Incidence of Paget's osteitis deformans.*

The separate and combined totals of cases associated with Paget's disease of bone are shown in Table VIII.

TABLE VIII.—*Osteogenic Sarcomata associated with Paget's disease.*

| | Males. | | | | | Females. | | | | |
|---|---|---|---|---|---|---|---|---|---|---|
| | 50–. | 60–. | 70–. | 80–. | Total. | 50–. | 60–. | 70–. | 80–. | Total. |
| BTR | 2 | 3 | 4 | 1 | 10 | 4 | 2 | 2 | 1 | 9 |
| BECC | | | | | 6 | | | | | 7 |
| Combined | | | | | 16 | | | | | 16 |
| Incidence in total cases in age group (34) | | | | | 47% | Incidence in total cases in age group (27) | | | | 59% |
| Incidence in combined series of 61 sarcomata (over 50 yrs.) | | | | | | | | | | 52·4% |

This table shows the approximately equal sex distribution of Paget's disease in cases of osteogenic sarcoma over 50 years of age, the condition being well advanced and active in about one half of the senile bone sarcomata encountered. On correcting these crude figures for the sex distribution of persons over 50 years (Males 46 per cent, Females 54 per cent), the totals in Table VIII would be modified to give : males, 16 sarcomata ; females, 14 sarcomata. There is again here a small relative male preponderance.

During examination of the BTR collection of specimens, evidence has been found suggesting extra-osseous connective tissue changes in Paget's disease. In one case (BTR/510, male aged 52) well advanced Paget's disease was associated with a soft tissue parosteal fibrosarcoma of the leg. Furthermore, although in the BTR juvenile group osteoblastic osteogenic sarcoma was seen three times as often as the osteolytic form, in the senile group this factor is reversed, the latter tumour being about three times as common.

Site analysis of the BTR series of sarcomata associated with Paget's disease fully confirms the point emphasized by Davie and Cooke (1937) that such growths are not infrequently multicentric in origin, and arise in the bone damaged by the osteitic process. Therefore, in general terms, the anatomical site distribution of the senile form of this sarcoma runs largely parallel with the skeletal distribution of Paget's disease, i.e. related to bone which is of membranous or periosteal origin, and with a relatively high site frequency in flat bones.

*Senile osteogenic sarcoma not related to Paget's disease.*

Two features have been noted in these cases :

A. Very occasionally there is evidence which suggests that the sarcoma is secondary to a pre-existing benign tumour, e.g. BTR/67, G. C.— male, aged 67 ; giant cell tumour of lower end of femur. BTR/230, C. H— male, aged 86 ; enchondroma of diaphysis of femur.

B. In some instances, particularly in femoral lesions, there has been radiographic and histological evidence of advanced osteoporosis. Histologically, as age advances, bone structure becomes more clearly defined, and both analytical and histochemical methods have shown tissue dehydration and relative loss of the mucopolysaccharide component of cartilage and inter-fibre cement substance. Histological examination of bone structure in the immediate vicinity of a number of senile osteogenic sarcomata, however, suggests that the continuation of this osteoporotic process may eventually lead to very extensive bone destruction via demineralization and disorganisation of the organic framework. Consequent vicarious formation of much new osteoid is also seen.

*Sex distribution.*

The sex distribution and relative tumour incidence for the combined BTR and BECC cases over 50 years of age are given in Table IX.

Examination of the combined BTR and BECC figures for cases over 50 years of age shows a crude sex ratio, (M/F.), of f. = 1·26, i.e. 5 males to 4 females. On correction, however, for population sex distribution, this ratio increases to f. = 1·54 i.e. 3 males to 2 females. This factor then approaches that found in the juvenile group, where f. = 2·0 (Table V). This male preponderance appears possibly to be reversed in the age period 50–60 years (f. = 0·75), and to be lower still in the preceding intermediate age group of 35–49 years, where f. = 0·48 (Table X).

TABLE IX.

| | Age. | | | | |
|---|---|---|---|---|---|
| *Males.* | 50–. | 60–. | 70–. | 80–. | Total. |
| Total tumours . . . | 9 | 15 | 7 | 3 . | 34 |
| % Tumours . . . | 26 | 44 | 21 | 9 . | 56 |
| % Population in age group . | 44·8 | 33·4 | 17·6 | 4·2 . | 46 |
| Tumour incidence $\frac{\% \text{ T.}}{\% \text{ P.}}$ . . | 0·6 | 1·3 | 1·2 | 2·1 . | 1·22 |
| *Females.* | | | | | |
| Total tumours . . . | 9 | 9 | 7 | 2 . | 27 |
| % Tumours . . . | 33 | 33 | 26 | 8 . | 44 |
| % Population in age group . | 40·8 | 32·9 | 20·5 | 5·8 . | 54 |
| Tumour incidence $\frac{\% \text{ T.}}{\% \text{ P.}}$ . . | 0·8 | 1·0 | 1·26 | 1·4 . | 0·8 |
| *Sex Factor*, corrected to f $= \frac{\text{T. incidence, M.}}{\text{T. incidence, F.}}$ . . | 0·75 | 1·3 | 0·95 | 1·5 . | 1·54 |

Note.—Per cent of tumours in each decade calculated upon basis of total tumours, (M. or F.) over 50 years = 100.

Per cent of population in each decade based upon proportions of male and female population in decade, of all persons over 50 years of age.

Totals in last column calculated from ratio :

$$\frac{\text{Per cent tumours male (or female) over 50 years.}}{\text{Per cent population male (or female) over 50 years.}}$$

TABLE X.—*Intermediate Age Group*—35–49 *years.*

| | Intermediate Age Group—35–49 years. |
|---|---|
| *Males.* | |
| Total tumours in period . . | 5 |
| % Total tumours in period . | 31·2% |
| % Total population in age period . | 50·5% |
| Tumour incidence. $\frac{\% \text{ T.}}{\% \text{ P.}}$ . . | 0·67 |
| *Females.* | |
| Total Tumours in period . . | 11 |
| % Total tumours in period . | 68·8% |
| % Total population in age period | 49·5% |
| Tumour incidence. $\frac{\% \text{ T.}}{\% \text{ P.}}$ . . | 1·4 |
| *Corrected Sex Factor.* | |
| f. $= \frac{\text{T. incidence, male.}}{\text{T. incidence, female.}}$ . . | 0·48 |

The inference from these figures is that the factor or factors which cause the relative male preponderance of osteogenic sarcomata in juveniles, are still operative in the senile group. It has already been suggested that this male frequency is related to the overall greater bone length growth of men, which feature may also basically influence the increased male incidence of Paget's disease in like manner. The data of Table VIII would support this assertion, but are not conclusive, since even after correcting for the population sex distribution at this age, the male relative increase is only very small. As in the juvenile group, so here also, there is some suggestion from the corrected age/sex distribution curve (Fig. 7) that the incidence of osteogenic sarcoma in later years rises somewhat more rapidly for women.

*Discussion—senile group of cases.*

On comparison, the sex incidence differences noted in senile cases of osteogenic sarcomata are absent in the groups of fibrosarcomata and lymphosarcomata of similar age of the several series considered above. In the BECC 1949 series of both of these latter tumours, the corrected sex factor (M./F) in respect of patients over 45 years of age was found to be 0·85 for lymphosarcoma and 1·05 for fibrosarcoma. In the larger American series of fibrosarcomata of Stout (1948) no details are given for the age/sex distribution ; it was however stated that the total group included 100 men and 106 women.

(*Note.*—Although lymphosarcoma usually shows a well marked male preponderance when all ages are combined, the BECC 1949 series records 34 males and 31 females over 45 years of age. On applying to these figures the appropriate correction for sex distribution in the population over 45 years, the ratio

$$\frac{\text{Tumour incidence, male}}{\text{Tumour incidence, female}} = 0{\cdot}85.)$$

These differences suggest that assuming the causative mechanism of these three forms of sarcoma to be of a similar nature, in the case of osteogenic sarcoma the relative frequency in persons over 50 years of age is markedly influenced by some added factor which is sex hormone dependant.

### SUMMARY AND CONCLUSIONS.

1. A series of 67 osteogenic sarcomata has been analysed for the factors of age and sex distribution and incidence. The resulting data have been supplemented by a further series of 73 osteogenic sarcomata reported by the British Empire Cancer Campaign (1949). These figures have been compared and contrasted with two American series, and also with groups of cases of primary chondrosarcoma, lymphosarcoma and fibrosarcoma.

2. The data presented shows that the incidence of osteogenic sarcoma is relatively higher amongst the population of England (S.W. Region) during the 7th and 8th decades than in the 2nd decade, as is most generally believed. In view of the small number of cases available in this series it is not clear as to the relative incidence in the 9th decade, although there is some suggestion that this may be still higher than in the two preceding periods. These latter features resemble the late age incidence trends in soft tissue fibrosarcoma. This latter tumour however does not show, in the series examined, the differential male preference which is seen in osteogenic sarcoma, both juvenile and senile.

3. The sex incidence of the cases of the age period under 35 years has been correlated with the known features of juvenile bone growth. It is suggested that the sex incidence differences are due to the characteristically earlier development of the female skeleton, and the greater total bone growth in length of the male.

4. The intermediate age group (35–49 years) provides too few cases for detailed treatment, but indicates the comparative rarity of bone sarcoma of this type during this period of life. In the very small number of cases considered there is reversal of the otherwise usual male predominance.

5. In the senile group (over 50 years) one half of all cases here reported were associated with Paget's osteitis deformans. After correction for differences in population age/sex distribution the male sex predominance is again found to be about the same order as that encountered in the juvenile group.

In addition to the original list of cases formerly studied by the author (1952), further material has been included here from the following sources, all being cases referred to the Bristol Bone Tumour Register :

| | |
|---|---|
| J. Bastow, Esq., F.R.C.S. | Nos. 516, 705. |
| Dr. S. Curwen, D.M.R.T. | No. 67. |
| A. L. Eyre-Brook, Esq., F.R.C.S. | Nos. 44, 450, 512, 754. |
| Dr. H. J. Gibson. | Nos. 522, 572. |
| Dr. R. C. Hadden. | Nos. 406, 414. |
| Dr. E. J. Harries. | No. 595. |
| H. K. Lucas, Esq., F.R.C.S. Ed. | Nos. 416, 423, 440. |
| Dr. E. M. Martland | No. 675. |
| W. R. Mitchell, Esq., F.R.C.S. | Nos. 691, 752. |
| J. A. Penrose, Esq., F.R.C.S. | No. 664. |
| K. H. Pridie, Esq., F.R.C.S. | Nos. 504, 538. |
| Dr. R. Sandry. | Nos. 737, 741. |
| J. B. Shield, Esq., F.R.C.S. | Nos. 579, 590, 592. |
| Dr. G. Stewart Smith. | No. 586. |
| Dr. A. L. Taylor | Nos. 428, 529. |
| Dr. J. C. Valentine. | Nos. 465, 588. |

The author wishes to record his thanks to the surgeons, radiotherapists and pathologists who have referred these cases, and have rendered invaluable assistance by the loan of clinical notes, skiagrams and pathological specimens. Thanks are also due to Mr. A. E. S. Roberts of the Medical Library, University of Bristol, for much help ; also to Dr. H. A. Sissons for his helpful criticism of this paper.

Fig. 4 is reproduced by kind consent of the author and publishers of the *Annals of Surgery* ; Fig. 9 has been copied by permission of the publishers. This courtesy is also acknowledged with thanks.

This present work, together with that of the Bristol Bone Tumour Register, has been generously aided by grants from the British Empire Cancer Campaign, and from the Cancer Research Fund of the University of Bristol.

### REFERENCES.

British Empire Cancer Campaign.—(1949) *Rep. Brit. Emp. Cancer Campgn*, **27**, 289.

CHRISTENSEN, F. C.—(1925) *Ann. Surg.*, **81**, 1074.

COLEY, B. L.—(1949) ‘ Neoplasms of Bone.’ New York (Hueber).

DAVIE, T. B. AND COOKE, W. E.—(1937) *Brit. J. Surg.*, **25**, 299.

DIGBY, K. H.—(1916) *J. Anat., London*, **50**, 187.

GESCHICKTER, C. F. AND COPELAND, M. M.—(1949) ‘ Tumours of Bone.’ 3rd. Ed. London (Lippincott).

GILL, G. G. AND ABBOTT, LE R. C.—(1942) *Arch. Surg.*, **45**, 286.

HARRIS, J. A., JACKSON, C. M., PATERSON, D. G. AND SCAMMON, R. E.—(1930) ‘ The Measurement of Man.’ Minneapolis (Univ. Minnesota Press).

PRICE, C. H. G.—(1952) *Brit. J. Cancer*, **6**, 46.

Registrar General.—(1953) *Statistical Review of England and Wales, for the year* 1952, Tables, Part II, Civil. London (H.M. Stationery Office).

SIMMONS, K.—(1944) Monogr. Soc. Res. Ch. Dev. 9. No. 1.

*Idem* AND GREULICH, W. W.—(1943) *J. Pediat.*, **22**, 518.

SHUTTLEWORTH, F. K.—(1938) Monogr. Soc. Res. Ch. Dev. 3. No. 5.

STONE, C. P. AND BARKER, R. G.—(1937) *Hum. Biol.*, **9**, 1.

STOUT, A. P.—(1948) *Cancer*, **1**, 30.

STRATZ, .—(1933) Quoted in “ Bone Growth in Health and Disease ” by H. A. Harris, p. 90. London (Oxford University Press).

TODD, T. W.—(1937) ‘ Atlas of Skeletal Maturation. St. Louis. (Mosby).

*Vital Statistics Rates in the United States*, 1900–1940—(1943) Edited by Linder, F. E. and Grove, R. D. Washington, D.C. (U.S.G.P.O.).